# Glutathione-Mediated Conjugation of Anticancer Drugs: An Overview of Reaction Mechanisms and Biological Significance for Drug Detoxification and Bioactivation

**DOI:** 10.3390/molecules27165252

**Published:** 2022-08-17

**Authors:** Agnieszka Potęga

**Affiliations:** Department of Pharmaceutical Technology and Biochemistry, Faculty of Chemistry, Gdańsk University of Technology, Gabriela Narutowicza Str. 11/12, 80-233 Gdańsk, Poland; agnieszka.potega@pg.edu.pl; Tel.: +48-58-347-15-16

**Keywords:** anticancer drugs, glutathione, mechanisms of glutathione conjugation reaction, detoxification, bioactivation

## Abstract

The effectiveness of many anticancer drugs depends on the creation of specific metabolites that may alter their therapeutic or toxic properties. One significant route of biotransformation is a conjugation of electrophilic compounds with reduced glutathione, which can be non-enzymatic and/or catalyzed by glutathione-dependent enzymes. Glutathione usually combines with anticancer drugs and/or their metabolites to form more polar and water-soluble glutathione S-conjugates, readily excreted outside the body. In this regard, glutathione plays a role in detoxification, decreasing the likelihood that a xenobiotic will react with cellular targets. However, some drugs once transformed into thioethers are more active or toxic than the parent compound. Thus, glutathione conjugation may also lead to pharmacological or toxicological effects through bioactivation reactions. My purpose here is to provide a broad overview of the mechanisms of glutathione-mediated conjugation of anticancer drugs. Additionally, I discuss the biological importance of glutathione conjugation to anticancer drug detoxification and bioactivation pathways. I also consider the potential role of glutathione in the metabolism of unsymmetrical bisacridines, a novel prosperous class of anticancer compounds developed in our laboratory. The knowledge on glutathione-mediated conjugation of anticancer drugs presented in this review may be noteworthy for improving cancer therapy and preventing drug resistance in cancers.

## 1. Introduction: The Place of Glutathione in Drug Metabolism

Living organisms are continuously and unavoidably exposed to xenobiotics, including anticancer drugs. Many of these compounds are lipophilic in nature, and the body can only expel them through chemical modifications, known as biotransformations. Generally, these occur by mechanisms conveniently categorized as phase I and phase II metabolic transformations, which act in a tightly integrated manner [1,2]. Phase I metabolism alters the chemical structure of the drug by ‘functionalization reactions’ which introduce a polar functional group onto the molecule. Thus, phase I reactions are usually oxidation, reduction, dealkylation, epoxidation, oxidative deamination reactions, etc., which are primarily catalyzed by enzymes of the cytochrome P450 (P450; E.C. 1.14.-.-.) superfamily [1,3]. These reactions typically result in metabolites that are more water-soluble than the parent compounds and still retain some of their pharmacological activity [4]. Phase II metabolism generally serves as a detoxifying step in the metabolism of drugs and other xenobiotics and involves ‘conjugation reactions’ that couple the drug and/or its phase I metabolite molecule with an activated conjugating molecule. Conjugation usually renders the compound pharmacologically inert, more polar, and water soluble, so it can easily be excreted out of the body. The most prevalent phase II metabolic pathways include glucuronidation, sulfation, and glutathione conjugation [1,5]. The concept of two phases can also be extended to phase III, which is the excretion of the resulting phase II metabolites (conjugates) [6].

Although metabolic transformations aim to inactivate drugs, some chemical modifications, both in phase I and II metabolism, may also lead to the formation of highly reactive species, i.e., reactive drug metabolites, which are more pharmacologically active and/or toxic in comparison to the original compound [4,7]. Therefore, early knowledge about pharmacological and/or toxicological effects of metabolites of drug candidates is extremely desired for assessing their bioavailability, activity, and safety profiles in humans. In this regard, much attention is also paid to understanding the mechanisms of reactive metabolite generation. 

This work is focused on the conjugation reaction of xenobiotics with the reduced tripeptide glutathione (GSH = L-γ-glutamyl-L-cysteinyl-glycine), which is a significant route of drug elimination in phase II metabolism in many species [8]. GSH conjugation reaction may be non-enzymatic (i.e., chemical), but it is significantly accelerated by various GSH-dependent enzymes [9,10], mainly a family of GSH S-transferase (GST; E.C. 2.5.1.18) enzymes [11]. A number of anticancer therapeutics are known to undergo GSH conjugation reaction [12,13,14,15,16,17]. GSH can combine with electrophilic anticancer drugs and/or their phase I metabolites to form less toxic compounds than parent compounds and more polar, water-soluble GSH S-conjugates (thioethers). Further, GSH S-conjugates are substrates for transporters (phase III) involved in the biliary and renal excretion, which facilitates their clearance from the body [18]. Thus, GSH plays a role in detoxification, decreasing the likelihood that a xenobiotic will react with molecular and cellular targets. This may affect the efficacy and interactions of a variety of anticancer interventions. Moreover, numerous studies support the view that, in some cases, GSH conjugation may also play an important role in bioactivation reactions as it is able to generate GSH S-conjugates that are more active than the original parent xenobiotic [18,19,20]. If these reactive metabolites interact with critical cellular macromolecules (i.e., proteins, nucleic acids), toxicity can be ensued. By participating in the formation of toxic metabolites of some anticancer drugs, GSH may also affect the cellular uptake of other agents. For this reason, knowledge of these interactions may be useful in designing combination therapy for neoplastic diseases. 

Given this background, the study on GSH conjugation is a crucial factor for the determination of drug therapeutic efficacy and potential toxicity. Depending on the structure and physico-chemical properties of the substrate, the conjugation reaction may proceed according to various mechanisms. In this review, I discuss some of them in the context of anticancer drugs. In addition, I also indicate the potential significance of GSH conjugation in the detoxification and bioactivation of clinically important anticancer drugs.

To continue the search for potential anticancer drugs, a completely novel class of acridine derivatives of extraordinary structures, unsymmetrical bisacridines (UAs), has been synthesized and developed by our research group [21,22]. They were characterized with respect to their physico-chemical properties [23] and the role of phase I and phase II metabolic transformations in their action [24,25]. UAs exhibited high cytotoxic activity against a lot of cancer cell lines and high anticancer efficacy against several types of human cancer xenografts in nude mice [26]. These are mainly cancers that are extremely resistant to chemotherapy and are usually characterized by increased activity of various GST isoenzymes relative to normal tissues. Preliminary studies with rat liver microsomal and cytosolic subfractions showed the ability of C-2028 (9-{N-[(imidazo [4,5,1-de]-acridin-6-on-5-yl)aminopropyl]-N-methylaminopropylamino}-1′-nitroacridine, a representative UA) to GST-mediated and/or direct GSH conjugation [25]. This finding may suggest an important role of detoxifying transformations in UA metabolism, the consequences of which for the anticancer and/or toxic activities of the compounds are not yet well understood. Herein, I would like to present some considerations on this issue and indicate the likely mechanism of UA-GSH S-conjugate formation.

## 2. Glutathione

### 2.1. Glutathione Structure as a Determinant of Its Biological Functions

Glutathione is the predominant intracellular thiol-containing tripeptide found in all animal tissues, plants, fungi, and some microorganisms [27,28]. Under physiological conditions, it is mainly present in the cytoplasm in the reduced form (GSH), which is also the biologically active form. GSH is less easily oxidized than its precursors, cysteine and γ-glutamylcysteine; the fully oxidized form with a disulfide between two identical GSH molecules (GSSG) represents less than 1% of the total GSH pool in the cell [29]. GSH concentration in human cells typically ranges from 0.1 to 10 mmol/L, being most focused in the liver (up to 10 mmol/L), spleen, kidney, lens, erythrocytes, and leukocytes [30,31], wherein its depletion and/or altered level are associated with various diseases, including cancer, cardiovascular, inflammatory, immune, metabolic, and neurodegenerative diseases [32]. Maintaining optimal GSH:GSSG ratios in the cell is critical to survival; hence, tight regulation of this system is imperative [33].

**Figure 1:**
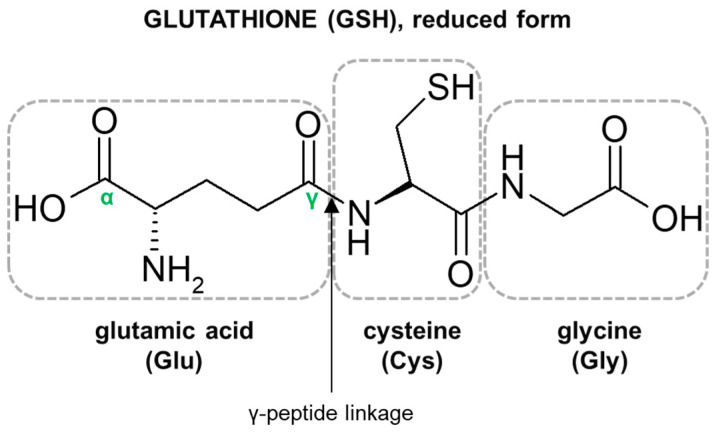
Structure of the tripeptide glutathione (GSH). Glutamic acid (Glu) is linked in a γ-peptide linkage (via its γ-carboxyl group) to cysteine (Cys), which in turn forms an α-peptide linkage with glycine (Gly).

The characteristic structural features of the GSH molecule (Figure 1) determine its many diverse biological functions. In contrast to an α-peptide linkage usually found in a number of endogenous peptides, the L-glutamic acid (Glu) and L-cysteine (Cys) of GSH are joined by an unusual γ-peptide linkage [28]. Such a bond promotes GSH resistance to hydrolysis by most intracellular aminopeptidases as well as provides for specificity in other GSH-enzyme interactions [34]. In turn, the activity of the high electron-donating sulfhydryl (thiol, -SH) group of Cys residue supports the reducing properties of GSH by way of a thiol-exchange system (-SH to -S-S-), enabling the participation of GSH in intracellular antioxidative and detoxifying reactions [35]. The reactivity of -SH is due to the thiolate anion (S^−^), the relative concentration of which is regulated by the acidity of thiol (pK_a_ = 9.2). At physiological pH, for every 100 GSH molecules in the -SH state, approximately 3.7 are in a thiolate form [36]. Due to the polarizability of the sulfur atom, GSH is a strong ‘soft’ nucleophile, and unlike other phase II enzyme cofactors such as uridine 5′-diphosphoglucuronic acid (UDPGA) and 3′-phosphoadenosine-5′-phosphosulfate (PAPS), it easily reacts with various ‘soft’ electrophiles [10], which may also be anticancer drugs. Net-negative charge of cysteinyl residue and overall GSH hydrophilicity greatly increase the aqueous solubility of the lipophilic moieties with which it becomes conjugated. GSH S-conjugates usually achieve a molecular weight higher than 300–500 g/mol (average molecular weight of GSH = 307.3235 g/mol) and are thus preferentially secreted via the biliary system. Then, the final cysteinyl conjugates are reabsorbed into the liver, from where they travel to the kidney for acetylation and excretion as a mercapturic acid [37]. 

### 2.2. Biological Functions of Glutathione

Reduced GSH has been adopted through evolution to perform multiple significant cellular functions in living organisms. It is responsible for the correct thiol–disulfide balance and the associated oxidation-reduction potential of cells [38]. The biologically important role of GSH is related to the possibility of regeneration of the -SH moieties of proteins, which counteracts the effects of oxidative reactions, inactivating cell proteins [39]. This compound is also involved in the reduction of ribonucleotides to deoxyribonucleotides, i.e., it has a direct impact on DNA biosynthesis and the related proliferation process [8]. Moreover, it mediates in the synthesis of proteins and in amino acid transport [40]. Further, GSH serves as a reservoir and transporter of cysteine [41], a regulator of calcium ion homeostasis [42], a versatile cofactor for many cytoplasmic enzymes [8], and it is a link in the mitochondrial mechanism to cell death [43]. 

In addition to the functions mentioned, GSH is an important component of the system that detoxifies both electrophilic xenobiotics and metabolically produced free radicals, i.e., reactive oxygen species (ROS), by the formation of GSH S-conjugates. Thus, it plays a central role in the protection of cells against a variety of exogenous and endogenous potentially harmful compounds [28,44]. The reactions to form GSH S-conjugates may be non-enzymatic, although they are greatly accelerated by GSH-dependent enzymes such as GSTs [11]. Hence, the effectiveness of the detoxification pathway depends upon the intracellular concentration of GSH, the presence of GSTs of appropriate specificity, and/or the capacity of the cell for rapid resynthesis of GSH [45]. Conjugation reactions of GSH with electrophilic compounds to GSH S-conjugates occur mainly in the liver, which exports GSH and has the highest GST activity [28]. Although by conjugation with GSH many compounds are rendered less toxic than the original parent xenobiotic, it has also been reported that some drugs, including those with anticancer activity, become more reactive following this reaction. Thus, GSH conjugation may also play an important role in drug bioactivation processes [18,19,20].

Overall, GSH acts in catalysis, metabolism, and transport. As knowledge about this tripeptide grows, it becomes more obvious that the GSH status is a highly sensitive indicator of cell functionality and viability. Moreover, although all GSH activities are important in the maintenance of normal cell homeostasis in living organisms, these can also constitute an advantage for cancer cells, allowing disease progression and resistance to therapy. 

### 2.3. Outline of the Regulation of Glutathione Levels in the Cell

**Figure 2:**
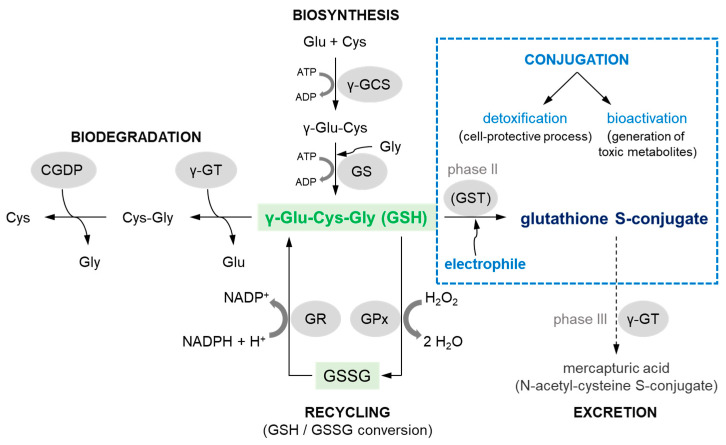
Comprehensive scheme of biosynthesis, biodegradation, and the most important reactions that reduced glutathione (GSH) may undergo in mammalian cells (scheme based on [47]). ADP = adenosine 5′-(trihydrogen diphosphate); ATP = adenosine 5′-(tetrahydrogen triphosphate); CGDP = cysteinylglycine dipeptidase; Cys = cysteine; Cys-Gly = cysteinylglycine; Glu = glutamic acid; Gly = glycine; GPx = GSH peroxidase; GR = GSH reductase; GS = GSH synthetase; GSSG = glutathione (oxidized form); GST = GSH S-transferase; H^+^ = positively charged hydrogen ion; H_2_O = water; H_2_O_2_ = hydrogen peroxide; NADPH/NADP^+^ = β-nicotinamide adenine dinucleotide 2′-phosphate, reduced/oxidized form, respectively; γ-GCS = γ-glutamylcysteine synthetase; γ-Glu-Cys = γ-glutamylcysteine; γ-GT = γ-glutamyl transferase.

Adequate intracellular GSH levels (and therefore the GSH:GSSG ratio) need to be carefully maintained in the cell. These are altered in biosynthesis, biodegradation, and consumption processes [44,46], which are presented schematically in Figure 2.

GSH biosynthesis, GSH biodegradation, and the recycling of component amino acids occur in the γ-glutamyl cycle, also known as the Meister cycle [48]. Briefly, GSH is synthesized intracellularly de novo from three precursor amino acids: L-glutamic acid (Glu), L-cysteine (Cys), and glycine (Gly). This requires the consecutive action of two enzymes, γ-glutamylcysteine synthetase (γ-GCS) and GSH synthetase (GS) (biosynthesis pathway in Figure 2). The bioavailability of Cys is rate-limiting for the synthesis of GSH [49]. GSH breakdown is initiated by the action of γ-glutamyl transferase (γ-GT), an enzyme attached to the external surface of certain cell membranes [30]. γ-GT transfers a Glu to other amino acids releasing cysteinylglycine (Cys-Gly), which in turn can be broken down by a cysteinylglycine dipeptidase (CGDP) to produce Cys and Gly [44] (biodegradation pathway in Figure 2). Reactions leading to a decrease in GSH levels in cells are also reactions with ROS and electrophilic compounds formed from xenobiotics that are considered to be toxic to the cell. In a recycling pathway GSH peroxidase (GPx), in concert with catalase and superoxide dismutase, detoxifies ROS such as hydrogen peroxide (H_2_O_2_) with GSH acting as an electron donor in the reduction reaction, producing GSSG as an end product. Within cells, GSH is regenerated from GSSG by GSH reductase (GR) in a process that requires reduced β-nicotinamide adenine dinucleotide 2′-phosphate (NADPH) [8].

**Figure 3:**
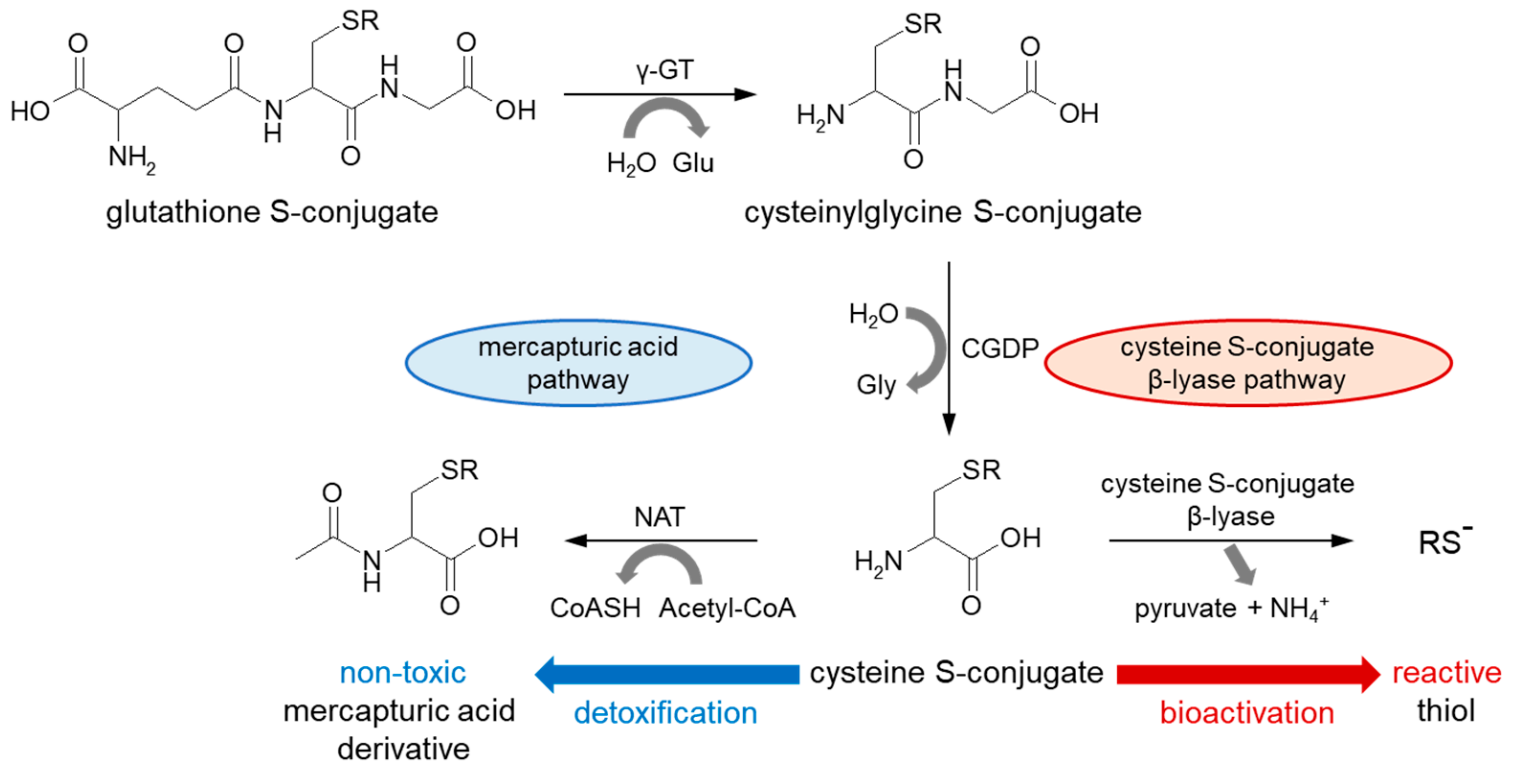
Metabolism of GSH conjugated product to mercapturic acid conjugated product in the mercapturic acid pathway and cysteine S-conjugate β-lyase-dependent bioactivation of cysteine S-conjugate. Acetyl-CoA = acetyl coenzyme A; CGDP = cysteinylglycine dipeptidase; CoASH = coenzyme A; Glu = glutamic acid; Gly = glycine; H_2_O = water; NAT = N-acetyltransferase; NH_4_^+^ = ammonium ion; γ-GT = γ-glutamyl transferase.

The nucleophilic conjugation of GSH with a wide spectrum of electrophiles (conjugation pathway in Figure 2) and transport of the corresponding GSH S-conjugates out of the cells have been shown to work as a system in the detoxification of xenobiotics, including anticancer drugs. The major components of this system include GSH, GSH-related enzymes (mainly GSTs), and GSH S-conjugate export pumps (GS-X pumps) [50]. Some researchers assume the further metabolism and/or excretion of GSH-labeled substances as phase III metabolic reactions (Figure 3). At the cell surface, the catabolism of GSH S-conjugate is initiated by the membrane-bound γ-GT, which removes (extracellularly) the GSH γ-glutamyl moiety and transfers it to appropriate acceptors. The resulting cysteinylglycine S-conjugate is further converted by cysteinylglycine dipeptidase (CGDP) to remove the glycyl group and produce the cysteine S-conjugate. Then, this compound re-enters the cell via various transport proteins where cytosolic N-acetyltransferases (NATs) create a mercapturic acid derivative of the xenobiotic (S-substituted N-acetyl-L-cysteine conjugate) [51,52]. The final product of the above reactions is generally non-toxic, more polar, and more water-soluble than the parent compound, and can be readily excreted outside the body through bile or urine [52]. Although, the presented mercapturic acid pathway and trafficking of mercapturic acid derivatives may vary with different compounds and species [18], GSH conjugation represents a cell-protective process through detoxification mechanism.

Alternatively, in recent years, it has been documented that the formation of glutathione S- and cysteine S-conjugates may be bioactivation events as the intermediate metabolites are toxic (i.e., cytotoxic, genotoxic, or mutagenic) [53,54]. For example, instead of intracellular acetylation, some cysteine S-conjugates (which usually contain a strong electron-withdrawing group attached to the sulfur atom) may be converted through β-elimination by cysteine S-conjugate β-lyases (present in the cytosol and mitochondria) to pyruvate, ammonium ion (NH_4_^+^), and an unstable and highly reactive thiol (RS^−^) [52,55]. This constitutes a bioactivation pathway. The sulfur-containing fragment further released in this process is presumably a sulfenic acid (RS-OH). Recent studies suggest that the coupling of enzymes of the mercapturic acid pathway to cysteine S-conjugate β-lyases may be more common in nature and more widespread in the metabolism of electrophilic xenobiotics than previously realized [18]. However, before addressing these aspects in the context of anticancer drugs, it is important to know the major mechanisms of their conjugation with GSH. 

## 3. Overview of Mechanisms of Glutathione Conjugation of Anticancer Drugs

**Figure 4:**
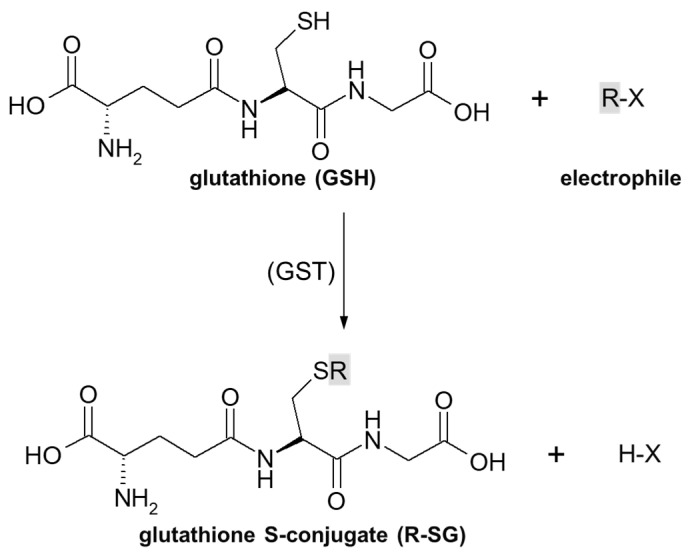
General scheme of glutathione (GSH) conjugation to a generic electrophile (R-X) to form the glutathione S-conjugate (R-SG). The reaction may be catalyzed by the enzyme glutathione S-transferase (GST).

As previously stated, the important part of the GSH molecule in terms of its chemical activity is the nucleophilic sulfhydryl (thiol, -SH) group of the cysteine residue. Generally, as illustrated in Figure 4, conjugation with GSH involves attachment of the xenobiotic molecule or its phase I metabolite (assigned as R-X) with this group to form the GSH S-conjugate (assigned as R-SG). In fact, the reactive nucleophilic species is the thiolate anion of GSH (GS^−^), whose concentrations at physiological pH are approximately 1% of GSH concentration (pK_a_ of GSH = 9.2) [38]. Due to the low electronegativity and high polarizability of the sulfur atom, GSH acts as a ‘soft’ nucleophile and, as such, can be used to sense the reactivity of ‘soft’ electrophiles (i.e., compounds that contain an acceptor atom with high polarizability, low electronegativity, and often unshared *p* or *d* valence electrons) [51]. Xenobiotics that are conjugated with GSH are either very electrophilic right away or are metabolized to electrophilic compounds. Some reactions of the tripeptide GSH with cellular electrophiles have spontaneous rates which vary considerably depending on the reactivity of the electrophile, and frequently, but not always, are catalyzed by various GST isoenzymes [34]. However, non-enzymatic reactions are usually much slower than those catalyzed by GSTs. 

There are several main mechanisms of GSH conjugation involved in detoxification and the potential bioactivation of anticancer drugs. Common GSH conjugation reactions are nucleophilic substitutions (including replacement of halogen atom or group of atoms with GSH molecule, the opening of the strained oxirane ring), nucleophilic displacement from saturated and aromatic carbon atoms, or nucleophilic addition to a polarized double or triple bond (Michael addition). The term ‘GSH S-conjugate’ usually refers to the product of the attack of GSH on an electrophilic carbon atom; however, GSH may also react with electrophilic oxygen, nitrogen, and sulfur atoms [56]. Below, I discuss certain mechanisms of these reactions for the selected known anticancer drugs. 

### 3.1. Nucleophilic Substitution

Substitution reactions, which involve the reaction of a nucleophile with an electrophile, are prevalent in physiological and metabolic processes, in the action of some drugs, and in the chemical synthesis of nearly all drugs [57]. Thus, nucleophilic substitution is also the basic and the most widespread mechanism of GSH conjugation of electrophilic compounds. It is observed in several families of anticancer drugs, including alkyl, allylic, benzylic, and aryl halides, nitrogen mustard derivatives, or platinum complexes [33]. Electrophiles are positively charged or have a polarized bond with a partial positive character. Electrophiles capable of undergoing substitution reactions have a leaving group—a species that can accept and stabilize the pair of electrons that make up the bond being broken [57]. 

**Figure 5:**
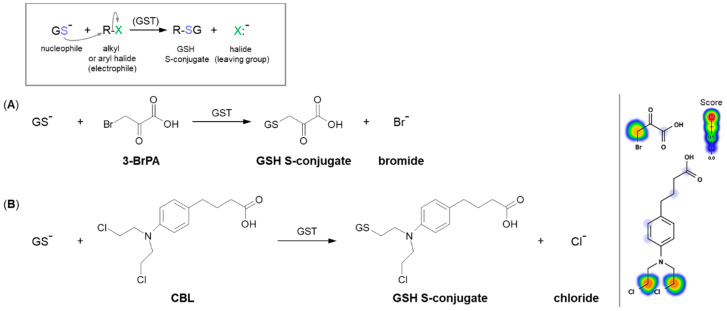
Reaction schemes of nucleophilic substitution of the thiolate anion of GSH (GS^–^) to the halogen atom (leaving group) in (**A**) 3-bromopyruvic acid (3-BrPA) [17] and (**B**) chlorambucil (CBL) [65]. The frame shows the general scheme of the GSH conjugation reaction. Predicted bioactive sites of GSH conjugation for anticancer drugs were obtained by XenoSite Reactivity Predictor available at https://swami.wustl.edu/xenosite/p/reactivity (accessed on 18 May 2022).

A common type of nucleophilic substitution reaction is the bimolecular nucleophilic substitution reaction, or S_N_2 reaction for short, where one atom or functional group is replaced with an electronegative GSH molecule (the frame in Figure 5). In this case, bond formation and bond breaking occur simultaneously and the leaving group tends to be a weaker base than the nucleophile. Halide ions, such as I^−^, Br^−^, and Cl^−^, are very good leaving groups and thus give fast reactions [58]. 

#### 3.1.1. Halogen Atom as a Leaving Group

The S_N_2 reaction mechanism of alkyl halide with GSH can be illustrated by the conjugation of 3-bromopyruvic acid (3-BrPA) with GSH (Figure 5A). 3-BrPA is the brominated derivative of pyruvic acid with proven anticancer activity against many different cancers in children and adults [59,60]. Like other α-bromoketones, it is widely known as a strong alkylating agent with a high affinity for protein binding as well as an antimetabolite [17]. Moreover, being an inhibitor of key glycolysis enzymes, including hexokinase II [61] and glyceraldehyde 3-phosphate dehydrogenase [62], 3-BrPA inhibits the growth of neoplastic cells that perform aerobic glycolysis known as the ‘Warburg effect’ [63]. Additionally, it also selectively blocks mitochondrial oxidative phosphorylation, angiogenesis, and energy production in cancer cells [63,64]. 

Based on a chemical view, the thiolate anion of GSH (GS^−^) easily attacks the carbon atom to which the bromine atom is attached. This reaction occurs because of the imbalance of the electron density between the carbon and halogen since it is a polar covalent bond. The more electronegative bromine atom pulls the electron density, thus making the carbon partially positively charged (an electrophilic center) and susceptible to a nucleophilic GS^−^ attack [66]. A bromine atom built into the structure of the 3-BrPA molecule is a good leaving group because the negatively charged bromine atom (bromide) is stable enough to exist on its own when it leaves the molecule. Hence, the conjugation to GSH does not require any prior metabolic activation of the parent compound. 3-BrPA was reported to form GSH S-conjugate both under GST catalysis and also in an enzyme-free system. Further, it is eliminated through the mercapturic acid synthesis pathway where it is excreted from the cell by ATP-binding cassette transporter proteins [17,67].

Another example of a similar S_N_2 reaction is the GSH conjugation of chlorambucil (CBL; the brand name: Leukeran) (Figure 5B). CBL is an alkylating agent approved for use in various malignant and non-malignant neoplasms, such as chronic lymphocytic leukemia [68], lymphosarcoma [69], and giant follicular lymphoma [70]. In the presence of GSH, CBL behaves as an efficient substrate for GSTA1-1 and GSTP1-1 isoenzymes [65,71]. As with the 3-BrPA, the formation of the corresponding GSH S-conjugate undergoes without prior CBL activation as the negatively charged chlorine atom (chloride) is a good enough leaving group. Moreover, kinetic data suggest that the rate-limiting of the catalytic reaction between CBL and GSH is the reaction product release [65]. 

#### 3.1.2. Tensioned Ring-Opening Reaction

**Figure 6:**
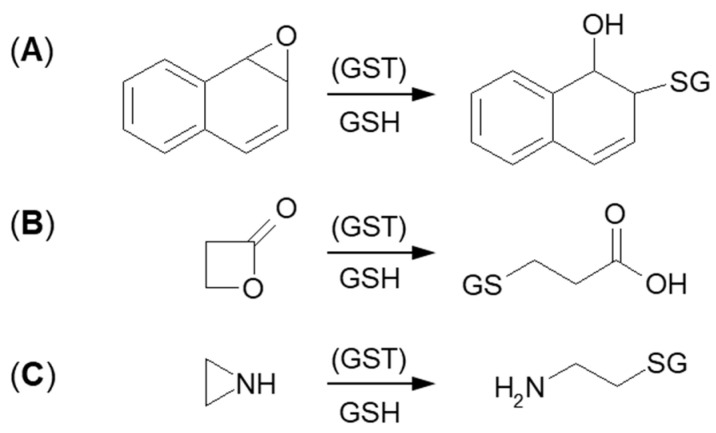
GSH conjugation via opening the tensioned ring of (**A**) epoxy, (**B**) lactone, and (**C**) aziridine.

The next type of GSH conjugation mechanism is the attachment of the thiolate anion (GS^–^) to the epoxide, four-membered lactone, or three-membered aziridine resulting in ring-opening (Figure 6). Such reactions take place quite easily because the rings are composed of three or four carbon atoms which are highly tensed and their energy is quite high. Additionally, such a process will be easier, if the tensed system includes a heteroatom, because it will inductively decrease the electron density on adjacent carbon atoms [72,73]. Under basic conditions, ring-opening occurs by an S_N_2 mechanism, and the less substituted carbon is the site of GS^–^ nucleophilic attack. The described transformation can take place stereoselectively, depending on the GST isoenzyme that catalyzes a reaction.

Formation of GSH S-conjugate, accompanied by the aziridine ring-opening, takes place, e.g., in the case of cyclophosphamide and thiotepa. Cyclophosphamide (CP; the brand name: Cytoxan) belongs to the alkylating agent and nitrogen mustard family of medications [74,75]. Its mechanism of action, quite similar to that of chlorambucil, relies on interfering with the duplication of DNA and RNA synthesis [76]. It is a chemotherapeutic approved to treat malignant lymphomas, neuroblastoma, multiple myeloma, leukemias, ovarian, breast, and many other cancers [77]. Such a wide spectrum of clinical uses makes it an essential component of numerous combination chemotherapeutic regimens. Moreover, CP is also used to suppress the immune system [78]. 

**Figure 7:**
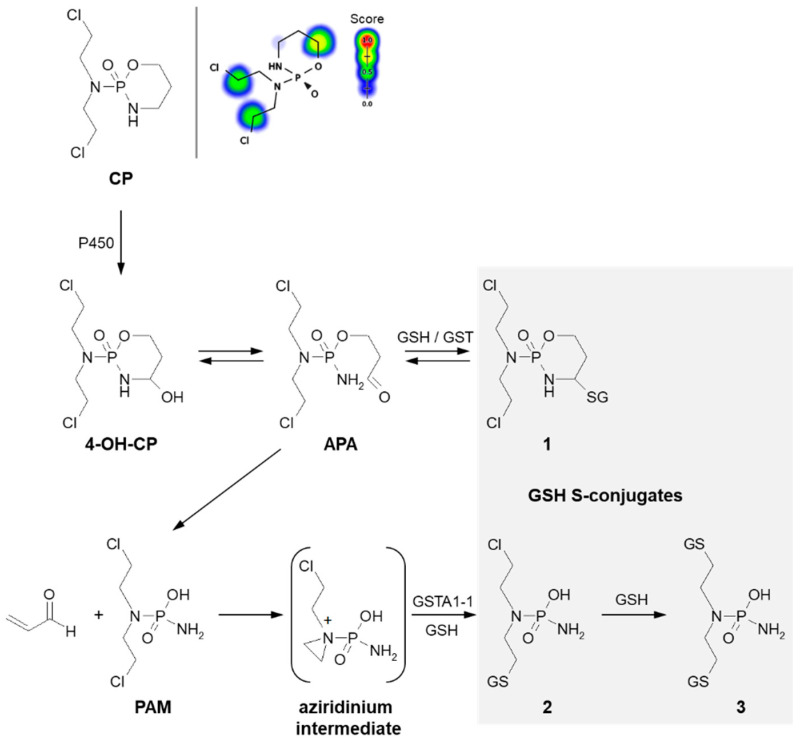
Scheme of cyclophosphamide (CP) activation and drug reaction with GSH [74]. Possible GSH S-conjugates: **1**: 4-monoglutathionyl cyclophosphamide, **2**: monoglutathionyl phosphoramide mustard, **3**: diglutathionyl phosphoramide mustard. 4-OH-CP = 4-hydroxycyclophosphamide; APA = aldophosphamide; PAM = phosphoramide mustard; P450 = cytochrome P450. Predicted bioactive sites of GSH conjugation for anticancer drug were obtained by XenoSite Reactivity Predictor available at https://swami.wustl.edu/xenosite/p/reactivity (accessed on 18 May 2022).

A prerequisite for conjugation with GSH is the presence of a reactive electrophilic metabolite(s). Unlike 3-BrPA and CBL, CP must undergo previous metabolic activation by hepatic microsomal mixed-function oxidases (i.e., P450s, mainly 2B6, 2C8, and 2C9) (Figure 7) [79,80]. The generally accepted mechanism for the generation of active metabolites of CP involves oxidation to the primary 4-hydroxycyclophosphamide (4-OH-CP) metabolite, which stays in tautomeric equilibrium with the ring-opened aldophosphamide (APA). Then, non-enzymatic cleavage of APA results in the formation of two toxic species—phosphoramide mustard (PAM) and acrolein. PAM is believed to unfold cell toxicity by DNA alkylation [81] while acrolein is held to be responsible for some aspects of host toxicity, such as hemorrhagic cystitis [82]. Therefore, GSH conjugation can result in the formation of three types of GSH S-conjugates, i.e., 4-monoglutathionyl CP (4-GSCP) (**1**), mono- (**2**) and diglutathionyl PAMs (**3**) [13,83]. The formation of 4-GSCP was found to be reversible, and by APA hydrolysis, PAM was formed. Thus, 4-GSCP can be considered a stable reservoir for the generation of PAM that subsequently undergoes two consecutive GSH conjugations. The reaction with nucleophilic GSH was established to proceed through the positively charged and highly polarized aziridinium ion (aziridinium intermediate) that can be opened in both the enzyme-catalyzed and the chemical reactions. On the other hand, the second GSH conjugation reaction was shown to occur through the direct displacement of chloride [13]. The 4-GSCP formation reaction can be catalyzed by various GSTs isoenzymes, whereas GST1A-1 has the greatest effect on the rate of monoglutathionyl PAM formation. Melphalan, mechlorethamine, ifosfamide, carmustine, lomustine, and nimustine are examples of other anticancer-active nitrogen mustard derivatives that react with GSH in a manner similar to that described for CP [13,80].

**Figure 8:**
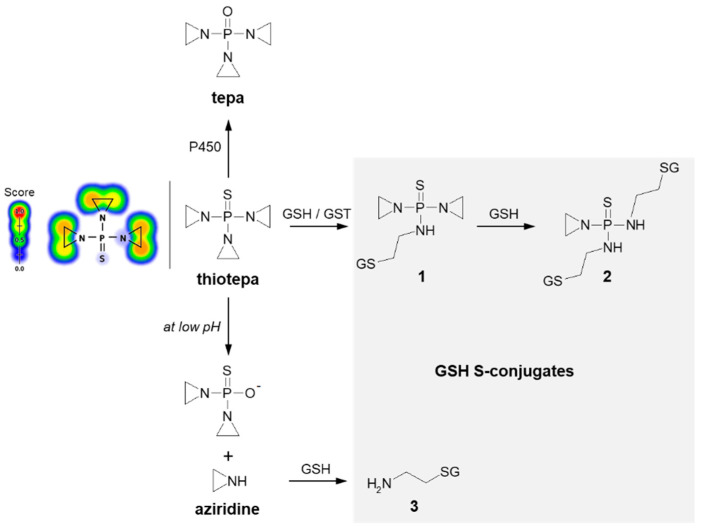
Scheme of thiotepa activation and drug reaction with GSH [89]. Possible GSH S-conjugates: **1**: monoglutathionyl thiotepa, **2**: diglutathionyl thiotepa, **3**: 2-aminoethyl glutathione. P450 = cytochrome P450. Predicted bioactive sites of GSH conjugation for anticancer drug were obtained by XenoSite Reactivity Predictor available at https://swami.wustl.edu/xenosite/p/reactivity (accessed on 18 May 2022).

Thiotepa (N,N′,N″-triethylenethiophosphoramide; the brand name: Tepadina) also acts as an alkylating agent [84]. Being active against a wide variety of cancers, it is commonly used in combination with other chemotherapeutic agents to treat ovarian cancer, bladder cancer, and breast cancer [85,86]. Chemically, it is an organophosphorus compound containing a four-coordinated phosphorus atom and three aziridine moieties (Figure 8) through which the drug probably induces crosslinks with DNA, interfering with DNA replication and cell division [87]. The major metabolite formed from thiotepa during P450-catalyzed transformation (i.e., desulfuration reaction) is N,N′,N″-triethylenephosphoramide (tepa) [88]. In turn, when reacting with GSH, the tensioned ring of thiotepa containing a nitrogen atom as a heteroatom is opened, leading to the formation of mono- (**1**) and diglutathionyl thiotepa (**2**). In the metabolic pathway of thiotepa, 2-aminoethyl GSH (**3**), which is the product of the direct GSH conjugation of aziridine, was also characterized. The results confirmed that only thiotepa but not its monoglutathionyl S-conjugate is a substrate for GSTs (mainly A1-1 and P1-1 isoenzymes). Moreover, the non-enzymatic reaction of the aziridinium moieties of thiotepa with GSH is strongly dependent on the pH, and the yield of the reaction is greatest at low pH [89].

#### 3.1.3. Glutathione Conjugation with an Atom Different from a Carbon Atom

**Figure 9:**
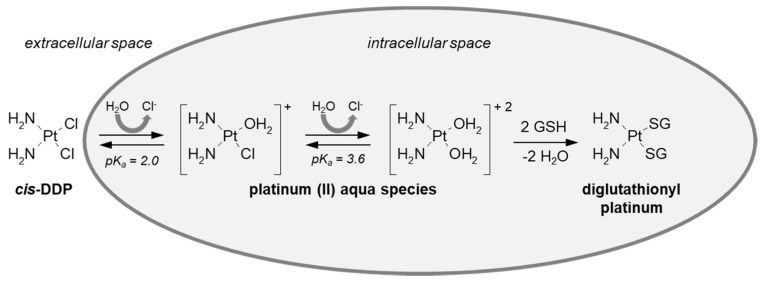
Reaction scheme of cisplatin (*cis*-DDP) conjugation with GSH.

GSH conjugation can also take place at an atom different from a carbon atom. Such a mechanism was described, e.g., for cisplatin (*cis*-diamminedichloroplatinum (II), *cis*-DDP). It is the most successful drug in the family of platinum-based anticancer compounds [90] and is extremely effective against a wide range of human solid neoplasms, including breast, testicular, and ovarian cancers [91,92,93]. The generally recognized mode of action of this non-organic drug is by binding to DNA (via cross-linking) and inhibiting its replication, which ultimately results in the death of the fastest proliferating cancer cells [94]. After *cis*-DDP enters the cell, there is a rapid replacement of one or both chloride ligands by water molecules (Figure 9) [52,95,96]. The resulting new platinum (II) aqua species are potent electrophiles and are thus predicted to readily react with a number of nucleophilic biological targets with the loss of the bound water molecules. Indeed, besides DNA, cisplatin is known to efficiently bind sulfur-containing proteins, which also perform a transport role for the drug, or various nucleophilic compounds, including thiols (e.g., GSH, N-acetylcysteine) [97,98]. Protein binding is related to the occurrence of drug resistance and toxic effects [99], which will be discussed further in this work. Ishikawa and Ali-Osman [100] first reported the formation of a diglutathionyl platinum conjugate in L1210 leukemia cells. Nagar et al. [101] found cisplatin-GSH S-conjugate in *Rattus norvegicus* and proposed the structure of the GSH-conjugated metabolite based on mass spectrometry.

There are several other platinum-based anticancer compounds such as carboplatin, picoplatin, and oxaliplatin, heavily applied in chemotherapy regimens [90], that conjugate to GSH in a similar manner to cisplatin [102,103]. No significant differences in their mechanism of action but several differences in effectiveness are observed. Some platinum derivatives are more tolerated by the human body than cisplatin [104]. 

#### 3.1.4. Aromatic Nucleophilic Substitution

**Figure 10:**
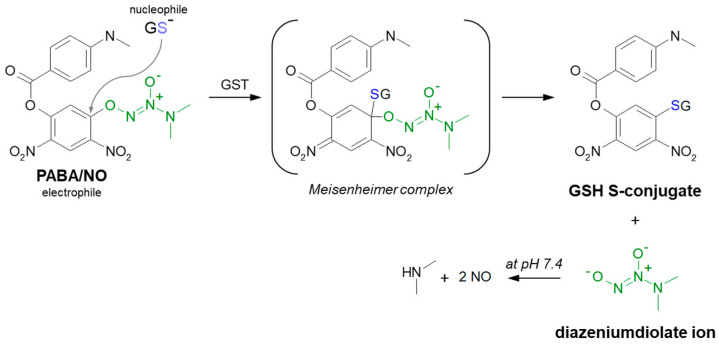
Scheme of the GST-catalyzed reaction of GSH with PABA/NO showing the formation of the Meisenheimer complex as the reaction intermediate [107]. A leaving group in PABA/NO molecule is marked in green.

Although aromatic rings are usually nucleophilic, some aromatic GST substrates can undergo nucleophilic aromatic substitution (S_N_Ar). In short, the aromatic ring is then not the attacking species but must be electron-poor (electrophilic). This is due to the negative inductive and mesomeric effects of its substituents. The rate-determining step of the reaction is an attack of the aromatic ring by the nucleophile, which disrupts aromaticity. The position in which the nucleophile attacks is defined by where the leaving group is, not by electronic and steric factors (i.e., no mix of *ortho*- and *para*- products as with electrophilic aromatic substitution) [57,58]. A typical illustration of the S_N_Ar reaction may be the substitution of GSH to 1-chloro-2,4-dinitrobenzene (CDNB), a model substrate for a GST conjugation activity [105]. The generally accepted mechanism of GS-DNB conjugate formation involves an addition-elimination sequence with a short-lived non-aromatic complex intermediate, the so-called Meisenheimer complex [106,107]. Among anticancer drugs, the S_N_Ar reaction is characteristic of GSH conjugation of PABA/NO. It was shown that this O^2^-aryl diazeniumdiolate produces anticancer effects comparable with cisplatin in a human ovarian cancer model grown in SCID mice and is also potent against the proliferation of the OVCAR-3 cell line [107,108,109]. GSH addition, selectively catalyzed by GST, proceeds with the formation of a Meisenheimer intermediate (Figure 10). PABA/NO was designed as a prodrug as the diazeniumdiolate ion leaving group subsequently spontaneously releases two moles of nitric oxide (NO) at physiological pH [108,109,110,111]. Therefore, within the GST-overexpressing cancer cells, the intracellular GSH is irreversibly consumed, and the NO generated this way could contribute to cancer therapy by inhibiting DNA synthesis, forming toxic reactive nitrogen/oxygen intermediates and inhibiting enzymes capable of preventing or repairing cellular damage [107].

**Figure 11:**
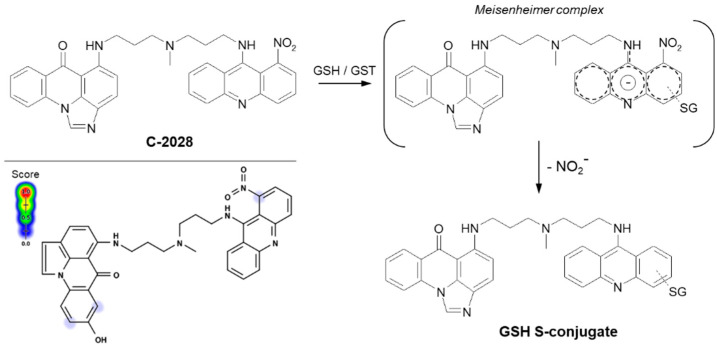
The proposed GSH-mediated metabolic pathway of C-2028, the representing anticancer unsymmetrical bisacridine derivative [25]. Predicted bioactive sites of GSH conjugation for anticancer agent were obtained by XenoSite Reactivity Predictor available at https://swami.wustl.edu/xenosite/p/reactivity (accessed on 18 May 2022).

Referring to the unsymmetrical bisacridines (UAs) studied in our group, the S-conjugate formation between GSH and UA derivative most likely occurs also according to the classical S_N_Ar mechanism. The general proposed mechanism of enzymatic GSH conjugation for a representative UA, compound C-2028, is shown in Figure 11. In the first step, GST-mediated deprotonation of the GSH molecule to the thiolate anion (GS^−^) should take place. We assume that the electronegative nitrogen atom of the amino (-NH-) functional group (electron-withdrawing group) decreases the electron density in the entire acridine ring and thus helps to stabilize a negatively charged intermediate, i.e., Meisenheimer complex [58], formed after the nucleophile attack. As a result, the nitro group may be easily removed from this transition state in the form of nitrite anion (NO_2_^−^) [112,113]. We provided evidence that the reaction did not require prior reduction of the nitro group, which would explain the observed lack of P450 involvement in the process [25]. Moreover, the results of our studies also indicated that 1-nitroacridine did not give any enzymatic GSH S-conjugate, while in the case of 1-nitro-9-aminoacridine and 1-nitro-9-methylaminoacridine, such products were detected (unpublished data). Thus, the presence of nitrogen atom from the amino group may be crucial in the GSH conjugation mechanism. Neither the structures of the proposed intermediate nor the GSH S-conjugate have been finally confirmed yet. 

### 3.2. Nucleophilic Addition (Michael Addition)

**Figure 12:**
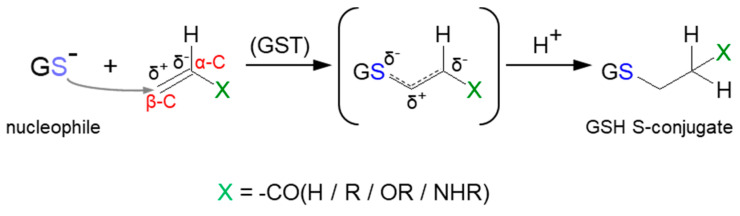
General scheme of Michael addition (or conjugate addition) to α,β-unsaturated compound with carbon−carbon double (C=C) bond.

Some reactions leading to the formation of GSH S-conjugates occur through Michael addition (or conjugate addition). Such a mechanism applies to α,β-unsaturated compounds (the so-called Michael acceptors) characterized by having carbon–carbon double (C=C) or carbon–carbon triple (C≡C) bonds with a strongly electron-withdrawing substituent(s) (e.g., a carbonyl, carboxyl, or nitro group) (Figure 12) [114]. This results in a polarizable electron density at the π bond, where the β-carbon atom (β-C) is positively polarized and becomes the preferred site of an attack of a soft nucleophile (Michael donor), e.g., the thiol group of cysteine in GSH. Although many of them can form thioethers non-enzymatically, GST-catalyzed Michael addition is much faster [10,115]. Compounds possessing Michael acceptor units feature a broad spectrum of bioactivity. They are considered to be particularly reactive and are thus capable of bonding with biological macromolecules [116].

Mitoxantrone (MTX; the brand name: Novantrone), a synthetic anthraquinone antineoplastic agent, is an example of a compound that undergoes GSH conjugation by Michael addition. MTX is a potent type II topoisomerase inhibitor that disrupts DNA synthesis and DNA repair in both healthy cells and cancer cells by intercalation between DNA bases [117]. It is commonly applied in the treatment of breast [118] and prostate [119] cancers, lymphomas [120], and leukemias, primarily acute myeloid leukemia [121], with excellent efficacy.

**Figure 13:**
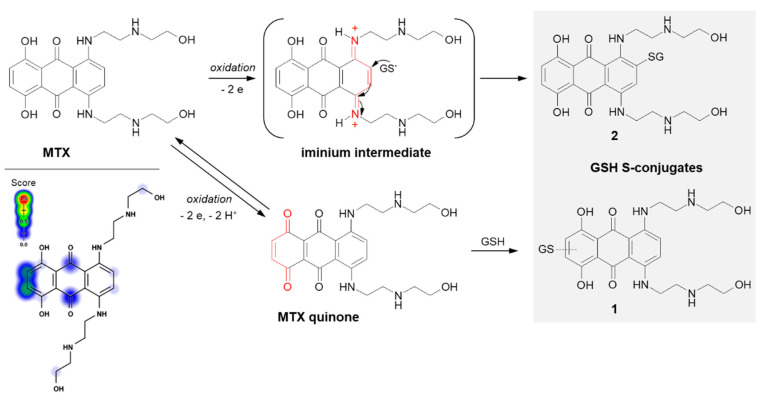
Scheme of mitoxantrone (MTX) activation and drug reaction with GSH [16,117]. The Michael acceptor moieties are marked in red. Predicted bioactive sites of GSH conjugation for anticancer drug were obtained by XenoSite Reactivity Predictor available at https://swami.wustl.edu/xenosite/p/reactivity (accessed on 18 May 2022).

In the case of MTX, at least two GSH conjugation pathways were identified (Figure 13). MTX is known to resist reductive enzymatic activation but is subject to facile oxidative enzymatic action [122]. The development of GSH-dependent resistance provides further evidence that the oxidative activation may be a relevant mode of drug action. The in vivo and in vitro studies of Mewes et al. [122] performed using minipigs, cultured rat hepatocytes, and human HepG2 hepatoma cells, respectively, revealed the formation of a major monoglutathionyl MTX (**1**) and its various degradation products. The ability of MTX to react with GSH enables the formation of an MTX quinone derivative by two-electron oxidation of the parent drug, which is in the form of a hydroquinone. Additionally, there are also data confirming the possibility of the formation of another MTX-GSH S-conjugate (**2**) [117]. Following the MTX enzymatic oxidation process within the aromatic ring containing polyamine side chains, the intramolecular Michael addition occurs. Presumably, the mechanism of this GSH conjugation reaction takes place via a labile iminium ion intermediate which activates the aromatic ring towards the attack of the external cellular nucleophile (e.g., GSH, DNA).

**Figure 14:**
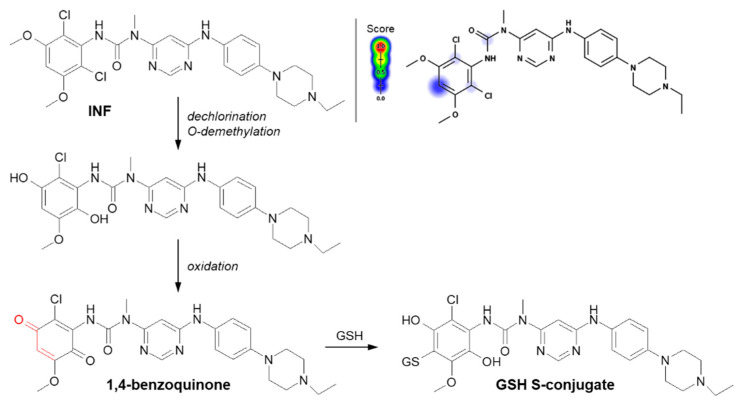
Reaction scheme of infigratinib (INF) conjugation with GSH [126]. A Michael acceptor moiety is marked in red. Predicted bioactive sites of GSH conjugation for anticancer drug were obtained by XenoSite Reactivity Predictor available at https://swami.wustl.edu/xenosite/p/reactivity (accessed on 18 May 2022).

Another example of a Michael addition-type reaction is infigratinib (INF, NVP-BGJ398). It is a novel small-molecule chemotherapeutic drug used for first-line treatment of advanced or metastatic cholangiocarcinoma (bile duct cancer). It was discovered that INF inhibits human fibroblast growth factor receptors (FGFRs), which are a family of receptor tyrosine kinases that may be upregulated in different cancer cell types [123]. For this reason, it is an investigational drug under development for the treatment of patients with various FGFR-driven diseases [124,125]. Al-Shakliah et al. [126] detected at least three GSH S-conjugate metabolites of INF using liquid chromatography ion trap mass spectrometry (LC-ITMS). As shown in Figure 14, the halogenated benzene ring of the INF structure undergoes metabolic bioactivation sequentially by dechlorination, O-demethylation, and oxidation to form the reactive 1,4-benzoquinone intermediate that is attacked by GSH [126,127].

**Figure 15:**
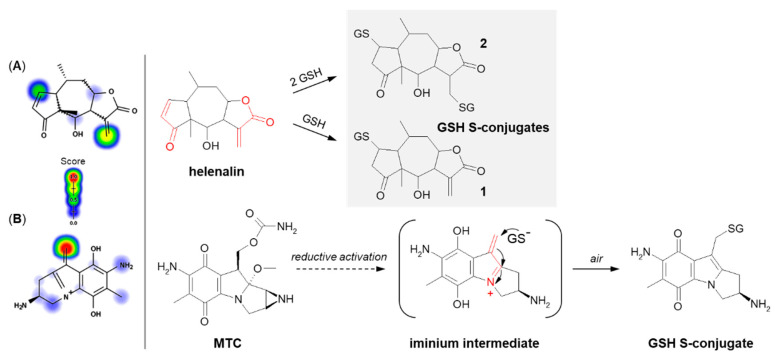
Reaction schemes of (**A**) helenalin [131] and (**B**) mitomycin C (MTC) [133] conjugation with GSH. The Michael acceptor moieties are marked in red. Predicted bioactive sites of GSH conjugation for anticancer drugs were obtained by XenoSite Reactivity Predictor available at https://swami.wustl.edu/xenosite/p/reactivity (accessed on 18 May 2022).

The next example is helenalin, a natural sesquiterpene lactone present in a large number of species mostly from the *Asteraceae* family, which has a variety of observed effects in vitro, including anti-inflammatory and anticancer activities [128,129,130]. As the compound structure contains reactive Michael acceptor systems (i.e., α,β-unsaturated ketone moiety and α,β-unsaturated lactone moiety), it is able to easily react with GSH [131]. The 2β-mono- (**1**) and 2,13β-diglutathionyl (**2**) conjugates of helenalin were shown to be formed by spontaneous Michael addition at physiological pH (Figure 15A). Interestingly, these were found to inhibit GST from horse liver, while free helenalin showed no inhibitory activity [132].

Mitomycin C (MTC) is a natural cytostatic antibiotic used as a chemotherapeutic agent by virtue of its anticancer activity. The mechanism of drug action is typical for that based on DNA alkylation [134,135]. Importantly, MTC requires previous activation via enzymatic reduction (bioreductive activation) to exert its biological effects. One-electron reduction steps to the corresponding semiquinone and then to hydroquinone initiates a cascade of consecutive reactions (i.e., spontaneous elimination of methanol from hydroquinone, elimination of the carbamate group, opening of the aziridine ring) that gives an unstable iminium intermediate which reacts with GSH through a Michael-type reaction [133,136] (Figure 15B). MTC was shown to form both mono- and diglutathionyl conjugates. It was also found that GSH itself did not reduce MTC, and unreduced drug did not form conjugates with GSH [137].

**Figure 16:**
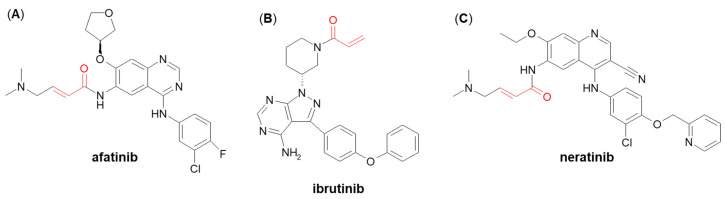
Chemical structures of (**A**) afatinib, (**B**) ibrutinib, and (**C**) neratinib. The Michael acceptor moieties are marked in red.

A few more examples of Michael acceptor-containing anticancer therapeutics metabolizing through GSH conjugation are afatinib, ibrutinib, and neratinib (Figure 16) [138,139]. They are all inhibitors of various tyrosine kinases and use their own Michael acceptor moiety for irreversible binding to a free cysteine residue of the targeted protein. In recent years, such a targeted covalent modification of regulatory proteins by Michael acceptors became recognized as a promising approach to drug discovery [140]. It can be expected that GSH plays an integral role in the clearance of these electrophilic drugs. Shibata and Chiba [138] showed that both afatinib and neratinib undergo extensive conjugation with GSH in buffer and cytosolic subfraction deriving from liver and kidney tissues, whereas ibrutinib has exhibited much lower degree of GSH/GST-dependent conjugation [138]. These findings may be helpful in optimizing pharmacokinetics in humans during the development stage of other targeted covalent inhibitors.

## 4. Possible Biological Consequences of Glutathione Conjugation of Anticancer Drugs

It is widely recognized that GSH conjugation of xenobiotics and conversion of thioethers to mercapturic acids is a biochemical defense of organisms against potentially harmful compounds [18]. In general, as a result of the detoxification process, many substances lose their toxic properties—completely or partially. However, in special cases, a diversion of this pathway (e.g., by the action of cysteine S-conjugate β-lyase) may lead to the bioactivation of some anticancer drugs rather than to detoxification [19,52], which means increasing their toxicity. Therefore, we can talk about the two directions of GSH conjugation of anticancer drugs, the possible consequences of which may be cancer treatment, resistance, or development. In this chapter, I would like to address this topic. 

### 4.1. Glutathione Conjugation as a Detoxification Reaction

The importance of GSH conjugation in the detoxification of a drug depends on the extent to which it is metabolized to reactive intermediates [34]. The reaction can be non-enzymatic (i.e., chemical) or enzyme-catalyzed, with GSTs playing the greatest role. The first is assumed to be particularly effective when soft and strong electrophiles are generated. In turn, the conjugation of soft but weak electrophiles (or hard electrophiles) requires enzymatic intervention to bring about effective GSH conjugation [10]. In order to deal with the wide variety of potential substrates, a multiplicity of GSTs exists—each tissue has its own collection, and each isoenzyme has a different substrate specificity. In humans, the highest cytosolic GST activity level is present in the liver, followed by the kidneys, lungs, and intestines [52,141]. There are many reports demonstrating that structurally different anticancer drugs form GSH S-conjugates through a GST-catalyzed process which are then degraded and removed from the body [18,51]. On the one hand, the GSH-related detoxification pathway reduces the drug reactivity and prevents further damage to the cellular macromolecules that could be caused by electrophilic metabolites and/or ROS. In some cases, conjugation with GSH can lead to the formation of up to 60% of the biliary metabolites [34]. The effectiveness of the detoxification pathway may depend on the intracellular concentration of GSH, the presence of GST of appropriate specificity, and/or the capacity of the cell for rapid resynthesis of GSH [45]. However, it should be remembered that the enhanced non-enzymatic GSH conjugation or overexpression of genes encoding particular GSTs in many cancers, relative to surrounding healthy tissues, were found to contribute to increased detoxification of anticancer drugs and, hence, to the development of drug resistance [50,142,143,144,145]. This frequently leads to a drop in the therapeutic effect of the chemotherapeutic and consequently to the decrease in its effectiveness during cancer therapy [141]. 

Cancer drug resistance is one of the problems usually associated with treatment with alkylating drugs [146,147,148]. For example, the detoxification of 3-bromopyruvic acid appears to be confirmed by a reduction in cancer cell viability due to a depletion in GSH levels in cells [67,149]. For chlorambucil, an inverse relationship was found between the GSH concentration and/or the GST activity and the number of DNA cross-links formed [150,151]. There are studies showing that both GSH levels and the presence of GSTs, and especially GSTA1-1, can influence the concentration of aziridinium intermediate formed from the cyclophosphamide and the toxicity of the compound [152]. GST-catalyzed GSH conjugation of thiotepa might be an important factor in the development of drug resistance since the overexpression of GSTP1-1, and to a lesser extent, GSTA1-1, is observed in cancer cells [89,153]. To overcome drug resistance, it is suggested that the moderate decline in the GSH level and/or GST activity would be a very effective approach for increasing the sensitivity of cancer cells to chemotherapy [147,154].

At this point, the phenomenon of ferroptosis should be mentioned. It is described as an intracellular iron-dependent and lipid peroxidation-driven regulated cell death pathway, which is different from other cell necrosis, apoptosis, and autophagy in morphology, biochemistry, and genetics [155]. Extensive studies suggest that ferroptosis correlates with cancer therapy resistance, and inducing ferroptosis has been demonstrated to reverse drug resistance [156]. Ferroptosis can be induced by agents causing the depletion of GSH (a cofactor of selenium-dependent GSH peroxidase 4 (GPx4)) or direct inhibition of GPx4 [155,156,157,158]. Thus, marked GSH consumption by electrophilic compounds may result in a reduction of GPx4 in drug-resistant cells, which provides new opportunities for cancer therapy. On the other hand, ferroptosis can be also involved in hepatotoxicity due to GSH depletion caused by drug overdose. For example, at nontoxic doses, the metabolite of acetaminophen (a drug used to treat pain and fever) is efficiently detoxified by GSH, forming an acetaminophen-GSH S-conjugate via Michael addition. However, when overdosed, it can cause potentially fatal hepatic centrilobular necrosis [159].

### 4.2. Glutathione Conjugation as a Bioactivation Reaction

In addition to its ‘classic’ function in anticancer drug detoxification, GSH together with enzymes of the GST family may be also involved in bioactivation reactions. In this case, the product of the initial conjugation is still reactive or even more reactive than the parent compound, and cellular macromolecules, within either cancer or normal cells of the host, become the main targets for their attack [19]. Different authors have reported some interesting findings on this topic [52,54,115,160,161]. Among the anticancer drugs, it is worth mentioning the examples of conjugates that are activated through (i) cysteine S-conjugate β-lyase, (ii) direct-acting GSH S-conjugates, (iii) conjugates that are activated through redox cycling, and (iv) conjugates that release the original reactive parent compound, i.e., GSH S-conjugates as targeted anticancer prodrugs [18,19]. These may be either toxic or pharmacologically active for the cells in which they were produced [52]. 

**Figure 17:**
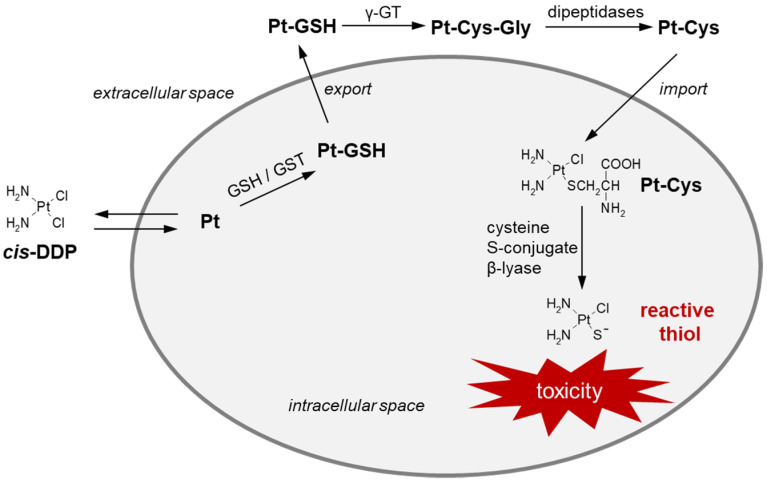
Cisplatin (*cis*-DDP) bioactivation through GSH S-conjugate (Pt-GSH) and cysteine S-conjugate (Pt-Cys) formation [18,52]. *cis*-DDP = cisplatin; GSH = glutathione; GST = glutathione S-transferase; γ-GT = γ-glutamyl transferase; Pt = platinum (II) aqua species.

The effectiveness of high-dose *cis*-DDP therapy is limited due to its side effects in the form of nephrotoxicity, ototoxicity, and neurotoxicity [162,163]. Damage of renal cells has been proven to be the result of cysteine S-conjugate β-lyase activity, which undergoes overexpression in this organ [164]. Figure 17 shows the activation pathway of *cis*-DDP to nephrotoxic species, which follows the mercapturic acid synthesis pathway, i.e., the conversion of *cis*-DDP to its GSH S-conjugate (Pt-GSH), and then to cysteine S-conjugate (Pt-Cys), the highly reactive and cytotoxic thiol version of cisplatin [18,52]. Cysteine S-conjugate β-lyase converts a drug intermediate into a thiol-reactive metabolite containing a Pt-SH (or Pt-S^−^) moiety that binds at thiophilic centers of mitochondrial proteins present in renal proximal tubule cells [164]. 

**Figure 18:**
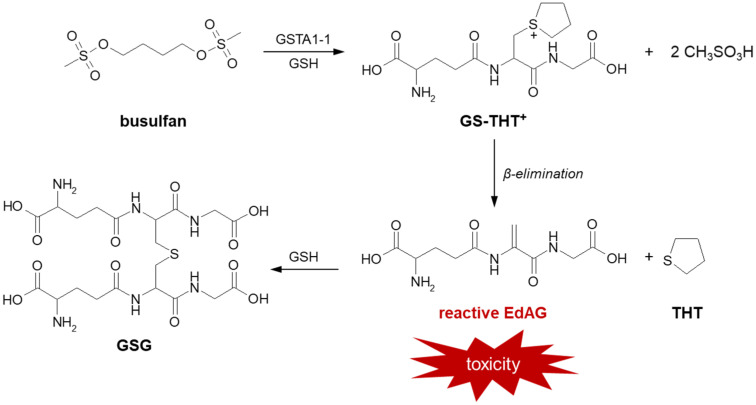
Formation of reactive busulfan metabolites in a GSH-dependent pathway [170]. EdAG = γ-glutamyl-dehydroalanyl-glycine; GSG = EdAG-GSH S-conjugate; GSH = glutathione; GST = glutathione S-transferase; GS-THT^+^ = glutathionyl-tetrahydrothiophene; THT = tetrahydrothiophene.

Further, there is strong evidence that GSH participates in the formation of a toxic metabolite of busulfan (the brand name: Busulfex) [18,19]. It is a cell-cycle non-specific bifunctional alkylating anticancer agent in the class of alkyl sulfonates. The drug is used in pediatrics and adults in combination with cyclophosphamide or fludarabine/clofarabine as a conditioning agent prior to bone marrow transplantation, especially in chronic myelogenous leukemia and other leukemias, lymphomas, and myeloproliferative disorders [165,166,167]. Toxicity related to busulfan treatment may include interstitial pulmonary fibrosis (the so-called ‘busulfan lung’), hyperpigmentation, seizures, hepatic (veno-occlusive disease) or sinusoidal obstruction syndrome [168]. It is believed that the above toxic effects are due to busulfan irreversible GSH conjugation catalyzed mainly by GSTA1-1 isoenzyme [169,170]. As a result of this reaction, presented in Figure 18, a positively charged product, glutathionyl-tetrahydrothiophene (GS-TH^+^), is formed. This ion is not sufficiently electrophilic to alkylate macromolecules, so it is then converted to γ-glutamyl-dehydroalanyl-glycine (EdAG) and the oxidation product of tetrahydrothiophene (THT) during β-elimination reaction. EdAG, which is an α,β-unsaturated dehydroalanyl analog of GSH, is considered to be a source of toxicity due to its chemical reactivity. Subsequently, it condenses with another GSH molecule via a Michael addition reaction to produce an oxidized lanthionine thioether (GSG). Unlike GSSG, GSG does not undergo reduction and reacts with protein thiol groups, resulting in the formation of irreversibly glutathionylated proteins. Irreversible glutathionylation has important implications for the mechanism of busulfan toxicity as it can lead to the loss of function of many proteins that are normally regulated by the reversible GSH conjugation reaction [171]. 

**Figure 19:**
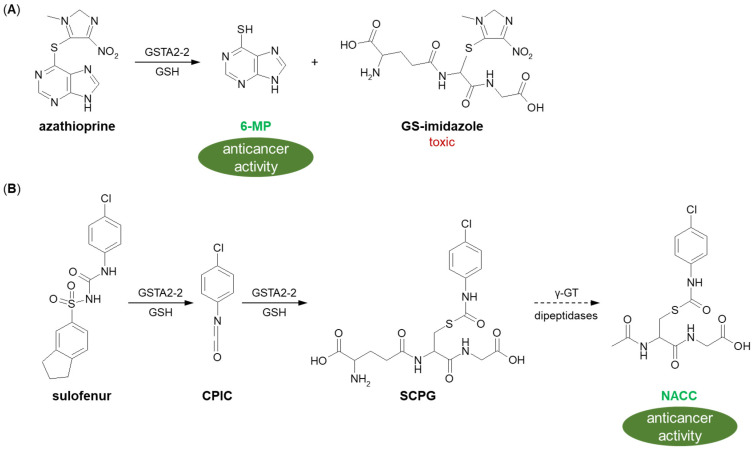
(**A**) Azathioprine [174] and (**B**) sulofenur [181] bioactivation to anticancer-active metabolites by GST-mediated GSH conjugation. 6-MP = 6-mercaptopurine; CPIC = *p*-chlorophenyl isocyanate; GSH = glutathione; GST = glutathione S-transferase; NACC = N-acetyl-S-(*p*-chlorophenylcarbamoyl) cysteine; SCPG = S-(N-*p*-chlorophenylcarbamoyl) GSH.

Drug precursors are pharmacologically inactive molecules in vitro that are converted into their active parent drugs in vivo after chemical modifications and/or enzymatic reactions [172]. They are often designed to improve the bioavailability of active drugs by increasing their amount in targeted cells and reducing off-target effects. A well-known example of a prodrug activated by GSH conjugation is azathioprine (brand name: Imuran). It is a methyl-nitroimidazole derivative of 6-mercaptopurine (6-MP) whose action of disrupting the formation of RNA and DNA in the cells assigns it to the purine analog and antimetabolite family of anticancer chemotherapeutics [173]. Enzymatic GSH conjugation of azathioprine, with the highest reaction efficiency observed for GSTA2-2, converts it into 6-MP and various methyl-nitroimidazole derivatives (Figure 19A) [174]. Released 6-MP incorporates into replicating nucleic acids, leading to an arrest of the de novo purine biosynthetic pathway [160,175]. In turn, GS-imidazole conjugate is believed to be the major route of azathioprine toxicity, especially hepatotoxicity, due to the high consumption of GSH, which is normally present in abundance in hepatocytes [176]. To sum up, the involvement of GSH in the metabolism of azathioprine is important for the toxification of the drug as well as the modulation of the therapeutic and toxic effects of the resulting 6-MP.

The category of targeted anticancer prodrugs also includes diarylsulfonylureas. A representative compound of this class, sulofenur (LY-186641) shows therapeutic efficacy against a wide variety of cancers [177,178]; however, it causes hemolytic anemia and methemoglobinemia at dose-limiting toxicities [179,180]. The anticancer and toxicological mechanism(s) of action of the drug is not well understood, but unlike other antineoplastic agents, sulofenur does not interfere with DNA, RNA, or protein synthesis, or with polynucleotide function. It undergoes metabolic transformation to form *p*-chlorophenyl isocyanate (CPIC), which could carbamoylate biological macromolecules directly or interact with GSH to give GSH S-conjugate (S-(N-*p*-chlorophenylcarbamoyl) GSH, SCPG) (Figure 19B) [181]. The resulting intermediate metabolite is further biotransformed via the mercapturic acid synthesis pathway to the corresponding N-acetylcysteine conjugate [N-acetyl-S-(*p*-chlorophenylcarbamoyl) cysteine, NACC], which expresses selective anticancer activity comparable to that observed for parent compound and has low toxicity [182]. The produced S-conjugates of GSH and cysteine are susceptible to thiol group exchange reactions and can therefore act as carbamoylating compounds for cellular macromolecules. Structural analogs of sulofenur have been studied and undergo similar metabolic pathways, which makes them promising anticancer drugs.

### 4.3. Anticancer Unsymmetrical Bisacridine Derivatives—Possible Biological Consequences of Glutathione Conjugation

The formation of GSH S-conjugate indicates the generation of reactive electrophilic metabolite(s) from a drug candidate that can bind to macromolecules of biological importance. When conjugation reaction is catalyzed by GST, the enzyme is a prime target for the attack. We predict that in vivo-formed GSH-conjugated metabolites of anticancer-active UAs (i.a., C-2028) would (i) lower the concentration of the nitroaromatic compound and its nitro group reduction derivatives, decreasing the overall toxicity of the chemotherapeutic agent (detoxification pathway) or (ii) serve as UA latent species (bioactivation pathway). This has not been fully recognized yet. Therefore, future works should focus on determining whether GSH S-conjugates of UAs exhibit any specific biological activity related to the toxicity and/or anticancer effects of these agents. Our preliminary studies revealed that the depletion of cellular GSH by buthionine sulfoximine (a specific inhibitor of γ-GCS) [183] or inhibition of GST activity by ethacrynic acid [184] decreased the sensitivity of Du-145 human prostate and H460 human lung cancer cell lines to C-2028, albeit to an extent dependent on the cell line (unpublished data). In line with these results, we can speculate on the GSH conjugation of UA as a bioactivation reaction.

One group of prodrugs may be molecules whose enzymatic activation occurs through a GSH-conjugate intermediate [185]. Compounds as NO donors, such as PABA/NO, are an important example. The presence of an easily reducible nitro group in the UA molecule suggests such an opportunity also among these potential anticancer drugs. Thus, the reduction of the nitro group may be a potential route of UA toxicity. In fact, UAs as NO-releasing agents would be able to induce differentiation and cell death in a variety of cancer cells through GSH consumption, DNA synthesis inhibition, and the inhibition of enzymes involved in the defense against cell damage. In this respect, the combination of UA molecules with the conventional anticancer treatment could be particularly effective. 

## 5. Concluding Remarks

In this work, the main mechanisms and the role of GSH conjugation in the biological action of several diverse anticancer chemotherapeutics have been discussed. The knowledge in these fields collected above was summarized in Table 1. The reactivity towards the thiol group of GSH (nucleophile) was predicted for most compounds (electrophiles) using the GSH model in the Xenosite Reactivity Predictor available at https://swami.wustl.edu/xenosite/p/reactivity (accessed on 18 May 2022), and these results matched the practical results found in the literature. The structure and physico-chemical properties of the substrate specify the type of GSH conjugation reaction and its subsequent consequences. Examples presented here clearly show that GSH/GST-mediated conjugation of anticancer drugs may represent a pathway for drug detoxification, cancer drug resistance, treatment, or therapy development. Thus, the GSH status of cancer or normal cells, the substrate selectivity of the GST, and the chemical properties of the GSH S-conjugate formed are among the strong determinants of the effectiveness and/or toxicity of the therapy. In conclusion, the knowledge about GSH conjugation of any anticancer drug may be exploited for the design and development of new anticancer drugs with better pharmacokinetic properties and lower overall toxicity, targeting specific cancers and avoiding the mechanisms of cancer cell resistance.

## Figures and Tables

**Table 1:** Overview of the mechanisms and the role of GSH conjugation of anticancer drugs discussed in this work.

Anticancer Drug	Type of GSH Conjugation Mechanism	The Crucial Role of GSH Conjugation in Drug Response
azathioprine	S_N_2/substitution of imidazole	bioactivation
3-bromopyruvic acid	S_N_2/substitution of halide	detoxification
busulfan	Michael addition	bioactivation (toxification)
chlorambucil	S_N_2/substitution of halide	detoxification
cisplatin	S_N_2/conjugation with an atom different from a carbon atom	bioacivation (toxification)
cyclophosphamide	S_N_2/tensioned ring-opening and substitution of halide	detoxification
helenalin	Michael addition	detoxification
infigratinib	Michael addition	detoxification
mitomycin C	Michael addition	detoxification
mitoxantrone	Michael addition	detoxification
PABA/NO	S_N_Ar	bioactivation
sulofenur	Michael addition	bioactivation
thiotepa	S_N_2/tensioned ring-opening	detoxification
unsymmetrical bisacridine	S_N_Ar (supposed)	ND

ND = not detected; S_N_2 = bimolecular nucleophilic substitution reaction; S_N_Ar = aromatic nucleophilic substitution.

## Data Availability

Not applicable.

## Abbreviations

3-BrPA, 3-bromopyruvic acid; 6-MP, 6-mercaptopurine; C-2028, 9-{N-[(imidazo [4,5,1-de]-acridin-6-on-5-yl)aminopropyl]-N-methylaminopropylamino}-1′-nitroacridine; CBL, chlorambucil; *cis*-DDP, cisplatin; CP, cyclophosphamide; Cys, L-cysteine; Glu, L-glutamic acid; Gly, glycine; GSH, L-γ-glutamyl-L-cysteinyl-glycine, glutathione (reduced form); GSSG, glutathione disulfide (oxidized form); GST, glutathione S-transferase; INF, infigratinib; MTC, mitomycin C; MTX, mitoxantrone; NO, nitric oxide; P450, cytochrome P450; ROS, reactive oxygen species; S_N_2, bimolecular nucleophilic substitution reaction; S_N_Ar, aromatic nucleophilic substitution; UA, unsymmetrical bisacridine.
